# Painless Circumcision

**Published:** 1891-02

**Authors:** G. Overall

**Affiliations:** Memphis, Tenn.


### PAINLESS CIRCUMCISION.

BY G. OVERALL, M. D., MEMPHIS, TENN.

For the past fifteen years I have performed circumcision quite frequently for the relief of various nervous disturbances. Since the introduction of cocaine I have used it almost exclusively in men, and frequently in children, by injecting it beneath the integument in the prepuce [having previously placed a rubber band around the penis half an inch or more back of the corona to limit its effect to the prepuce.]

The pain attending the introduction of the needle into the sensitive skin has been a serious objection in men, and almost as bad in children as the operation itself. Then I have had various postponements, and often complete abandonement of it, because I could not promise "that it would not hurt." Now I can promise an operation where a child would not even know it until it was performed.

I do not want my patient to see the operation, and in the case of a child I conceal the instruments. I then place the patient upon his back and lay a small pillow on his chest so that he cannot see over it. I then adjust the rubber band, take a freshly prepared thirty per cent. solution of cocaine and inject with a small blunt-pointed syringe a few drops into the preputial orifice, at the same time I hold the end of the prepuce with my left hand, to prevent the escape of the fluid. I then press upon the fluid with my right hand to enable it to come in contact with the entire mucous membrane. I then introduce carefully my hypodermic needle through the preputial orifice and penetrate the mucous membrane and inject a few drops of cocaine. I then move it to another part and repeat the injection. It necessarily requires caution to prevent puncturing the integument, which would cause pain. I operated upon a child six years old, very small, nervous, and excitable, while he was discussing with his mother the kind of toys he would get for Christmas. I also operated upon a boy fourteen years old that came from an adjacent town [using silk-gut ligature], he did not feel the slightest prick of the needle. He returned home the same evening and recovered without a bad symptom. It is always better to use a ligature that does not require to be removed.—*Medical Record.*

